# Comparative research on efficacy of needle grasper and mobile retractor-assisted laparoscopic high ligation of hernia sac in treatment of pediatric indirect inguinal hernia

**DOI:** 10.12669/pjms.42.8.15953

**Published:** 2026-08

**Authors:** Hanliang Jiao, Ruohan Wang, Zheng Dong, Baorui Wang, Shipeng Zhao

**Affiliations:** 1 Hanliang Jiao, Department of 2^nd^ General Surgery, Hebei Children’s Hospital, Shijiazhuang 050000, Hebei, China; 2 Ruohan Wang, Department of 2^nd^ General Surgery, Hebei Children’s Hospital, Shijiazhuang 050000, Hebei, China; 3 Zheng Dong, Department of 2^nd^ General Surgery, Hebei Children’s Hospital, Shijiazhuang 050000, Hebei, China; 4 Baorui Wang, Department of 2^nd^ General Surgery, Hebei Children’s Hospital, Shijiazhuang 050000, Hebei, China; 5 Shipeng Zhao Department of Colorectal Surgery, Hebei Medical University Third Hospital, Shijiazhuang 050000, Hebei, China

**Keywords:** Inflammatory Factors, Mobile Retractor, Laparoscopic high ligation of the hernia sac, Needle Grasper, Pediatric Indirect Inguinal Hernia

## Abstract

**Objective:**

To compare the clinical efficacy of needle grasper and mobile retractor-assisted laparoscopic high ligation of the hernia sac in the treatment of pediatric indirect inguinal hernia.

**Methodology:**

A retrospective analysis was conducted on 531 children with unilateral primary indirect inguinal hernia at Hebei Children’s Hospital who were admitted from January 2021 to December 2025. These children were divided into the grasper group(n=198) and the retractor group (n=333) according to the main instruments used for dissecting and ligating the neck of hernia sac during the surgery. Specifically, perioperative indicators, levels of inflammatory factors, complications, recurrence rate and other indicators were compared between the two groups.

**Results:**

The needle grasper group showed a significantly shorter operation time than the retractor group (27.74±9.60 min vs. 29.94±7.49 min, P=0.004) and the pain score at 24 h postoperatively was lower (3.26±0.67 vs. 3.99±0.80, P<0.001), along with a smaller increase in inflammatory factors such as IL-6 and CRP postoperatively (P<0.05). Additionally, no statistically significant differences were observed in intraoperative blood loss, incidence of early complications, detection rate of contralateral occult hernia and long-term recurrence rate between the two groups (P>0.05).

**Conclusion:**

Both needle grasper and mobile retractor-assisted laparoscopic high ligation of the hernia sac are safe and effective in the treatment of pediatric indirect inguinal hernia. However, needle graspers are superior in shortening the operation time and mitigating postoperative pain and inflammatory response, demonstrating better minimally invasive performance.

## INTRODUCTION

Pediatric indirect inguinal hernia is one of the most common congenital disorders in childhood, the etiology of which lies in the non-closure of the processus vaginalis and surgical intervention is the only effective method to cure the disease.^1^,^2^ With the rapid development of concepts and techniques of minimally invasive surgery, laparoscopic high ligation of the hernia sac (LHHS) has become one of the mainstream surgical methods for treating pediatric indirect inguinal hernia due to its remarkable advantages, such as small incisions, less pain, rapid recovery and the ability to simultaneously detect and treat contralateral occult hernias.^3^

In the specific operation of LHHS, how to safely, accurately and efficiently separate and ligate the neck of the hernia sac is a key step for the success of the operation. Combined with extraperitoneal injection of normal saline, needle graspers can bluntly and sharply dissect tissues, quickly separate the hernia sac without damaging the vas deferens and spermatic vessels. However, it requires a high level of spatial perception and fine operation from the surgeons.^4^ By contrast, mobile retractors can only navigate through the extraperitoneal space with their sharp tips. If the hernia sac is wide, it can make the procedure challenging, thus increasing the likelihood of penetrating the hernia sac and posing a risk of injury to the vas deferens and spermatic vessels.^5^

Despite the wide application of both auxiliary tools in clinical practice, the comparative research on their effectiveness in LHHS remains relatively limited and the conclusions are not completely consistent. In this regard, it is of great significance in clarifying the differences between the two instruments in terms of operation time, intraoperative blood loss, thoroughness of hernia sac dissection, risk of collateral damage to the vas deferens, incidence of postoperative complications and learning curve for optimizing surgical processes and improving surgical safety and standardization. Given this, this study is designed to compare the clinical efficacy of needle grasper vs. mobile retractor-assisted LHHS in the treatment of pediatric indirect inguinal hernia through retrospective analysis and to systematically evaluate the differences between the two methods in terms of intraoperative indicators, postoperative recovery and long-term follow-up results, so as to provide objective and reliable reference for the rational and personalized selection of auxiliary instruments in clinical practice.

## METHODOLOGY

This study adopted a retrospective cohort design. Specifically, data was collected through the hospital medical record system, children diagnosed with unilateral primary indirect inguinal hernia who underwent LHHS at Hebei Children’s Hospital between January 2021 to December 2025 were consecutively included. Based on the primary auxiliary instrument used for dissecting the neck of hernia sac during surgery, these children were divided into the grasper group (n=198, who underwent dissection and ligation of the hernia sac using a 2 mm needle grasper) and the retractor group (n=333, who underwent laparoscopy using a 2 mm mobile retractor).

### Ethical approval:

The study was approved by the Institutional Ethics Committee of Hebei Children’s Hospital (No.: 2022-09-18; Approval date: December 01, 2025), and written informed consent was obtained from guardians of all participants prior to their inclusion in the study..

### Inclusion criteria:

- Children aged six months to 12 years.
- Children with a definite clinical diagnosis of unilateral indirect inguinal hernia.
- Children receiving surgical treatment for the first time.
- Children definitely diagnosed via ultrasound preoperatively.
- Children with complete clinical data and follow-up records.

### Exclusion criteria:

- Children with incarcerated hernia, recurrent hernia, sliding hernia, or other abdominal diseases requiring concurrent surgery.
- Children with a history of surgery in the same inguinal region.
- Children accompanied by severe cardiac or pulmonary dysfunction, coagulation disorders, or other surgical contraindications.
- Children with conversion to open surgery.

### Surgical method:

Both groups received standardized treatment immediately after admission. All surgeries were performed by the same experienced pediatric surgical team, using the same general anesthesia and endotracheal intubation management. The child was placed in a supine position with the head lower than the feet. A 1 cm incision was made at the umbilical margin to establish a CO pneumoperitoneum (pressure: 8-10 mmHg; flow rate: 2-3 L/min) and a 5 mm 30° laparoscope was inserted for exploration. Under direct visualization with the laparoscope, a 2 mm puncture site was created at the surface projection of the internal ring on the affected side, allowing for the insertion of the operative instruments (grasper or retractor). The following operation was performed for the grasper group: A 2 mm needle grasper was inserted through the working port and the neck of the hernia sac (internal ring) and the medial structures, including the vas deferens and spermatic vessels, were identified within the abdominal cavity.

The blunt and sharp dissection was performed using the grasper in the loose space between the lateral peritoneum of the hernia sac neck and the vas deferens and spermatic vessels. After freeing the inner half of the hernia sac neck, the peritoneum was penetrated and a suture was left in the abdominal cavity. Later, the needle was withdrawn extraperitoneally to the site of insertion and the outer half of the hernia sac was similarly dissected. Afterward, the grasper was then re-inserted into the abdominal cavity to grasp the suture left inside and pull it out of the body, forming a complete external ligation loop. The suture was cut in the middle to create a double strand and the gas and fluid were expelled from the inguinal region and scrotum. Finally, the suture was tightened externally to ensure complete closure of the peritoneum at the internal ring under direct visualization, followed by tying two surgical knots, which were buried in the extraperitoneal space. By contrast, the following steps were taken for the retractor group: A 2 mm mobile retractor was inserted through the working port. With the tip of the retractor positioned on the medial side of the hernia sac neck, sharp dissection was performed between the peritoneum and the vas deferens and spermatic vessels (for a large hernia sac, gentle traction was applied to the affected testis to create counter-tension). After the tractor was maneuvered half a circle, the suture was left inside the abdominal cavity and the tractor was then withdrawn extra peritoneally to the site of insertion.

Similarly, the retractor was maneuvered half a circle extra peritoneally along the outer side of the internal ring before entering the abdominal cavity through the exit of suture. Afterward, the suture left inside the abdominal cavity was grasped and pulled out of the body using the retractor, so as to form a complete external ligation loop. Later, the suture was cut in the middle to create a double strand and the gas and fluid were expelled from the inguinal region and scrotum. Subsequently, the suture was tightened externally to ensure complete closure of the peritoneum at the internal ring under direct visualization, followed by tying two surgical knots, which were buried in the extraperitoneal space. Furthermore, both groups were examined for the contralateral internal ring and any occult hernia found was handled simultaneously. Finally, the pneumoperitoneum was released and the incision was closed.

### Observation indicators:

The following data were collected from the two groups for comparison: baseline characteristics, including age, gender, side of hernia and weight. Intraoperative indicators: Operation time (from skin incision to closure), intraoperative blood loss (estimated using gravimetric method or suction volume) and incidence of contralateral occult hernias. Postoperative indicators: Hospital stays, pain scores at 24 h postoperatively (via the FLACC scale) and incidence of early complications (e.g., scrotal emphysema/hematoma, incision infection). Inflammatory response indicators: 3 mL of fasting venous blood was collected from the children one day preoperatively, 24 hours postoperatively and 48 h postoperatively, so as to objectively assess the stress response induced by surgical trauma. In the meantime, serum levels of interleukin-6 (IL-6) and tumor necrosis factor-alpha (TNF-α) were measured using enzyme-linked immunosorbent assay (ELISA) and serum levels of C-reactive protein (CRP) were measured by immunoturbidimetry. Additionally, the occurrence of late complications such as recurrence, testicular atrophy and iatrogenic cryptorchidism was recorded through outpatient follow-up or phone consultations, with a follow-up period of ≥12 months.

### Statistical analysis:

The software SPSS 25.0 was used for data analysis. Measurement data following normal distribution were expressed as mean±standard deviation (*x̄*±*s*) and inter-group comparisons were conducted using independent samples t-tests; and measurement data not following normal distribution were expressed as median (interquartile range) [M(IQR)] and the Mann-Whitney U test was adopted for inter-group comparisons. Count data were expressed as cases (percentage) [n (%)], with inter-group comparisons performed using the χ² test or Fisher’s exact test. P<0.05 was considered statistically significant.

## RESULTS

A total of 531 children were included in this study, with 198 cases in the grasper group and 333 cases in the retractor group, respectively. There were no statistically significant differences in baseline characteristics such as age, gender distribution, side of hernia (left/right), weight and ASA grade between the two groups (P>0.05), indicating comparability Table-I.

**Table-I:** Comparison of baseline characteristics between the two groups [*x̄*±*s*/ n(%)].

Indicator	Grasper Group (n=198)	Retractor Group (n=333)	t/χ² value	P value
Age (years)	2.32± 1.48	2.23 ±1.50	t=-0.687	0.492
Gender (male)	162 (81.82)	285 (85.59)	χ²=1.323	0.250
Side of hernia (left)	92 (46.46)	140 (42.04)	χ²=0.987	0.320
Weight (kg)	16.79± 2.18	16.88 ± 2.03	t=-0.352	0.725
ASA Grade I	185 (93.43)	305 (91.59)	0.592	0.442

In terms of operation time, the grasper group reported significantly shorter times than the retractor group, with statistically significant differences (P<0.001). Both groups had minimal intraoperative blood loss, with no statistically significant inter-group differences (P>0.05). For the detection of contralateral occult hernia, the two groups exhibited a similar rate, with no statistically significant differences (P>0.05). Additionally, no significant differences were noted in postoperative hospital stays between the two groups. As for the time to first ambulation and pain scores at 24 hours postoperatively, the grasper group scored lower than the retractor group and the differences were statistically significant (P<0.05) Table-II.

**Table-II:** Comparison of perioperative indicators between the two groups.

Indicator	Grasper Group (n=198)	Retractor Group (n=333)	Statistics	P value
Operation time (minutes)	27.74± 9.60	29.94± 7.49	2.933	0.004
Intraoperative blood loss (ml)	10.18±3.06	10.40±3.42	0.754	0.451
Detection rate of contralateral occult hernia	28 (14.14)	51 (15.32)	0.135	0.713
Pain scores at 24 hours postoperatively (minutes)	3.26±0.67	3.99 ± 0.80	10.749	<0.001
Time to first ambulation (hours)	5.48±0.58	5.88±0.81	6.001	<0.001
Postoperative hospital stay (day)	3.92± 0.76	4.01±0.82	1.249	0.212

No significant differences were observed in baseline levels of IL-6, TNF-α and CRP between the two groups preoperatively (P>0.05). The grasper group showed a significantly lower increase in serum IL-6 and CRP levels than the retractor group at 24 hours and 48 hours postoperatively, with statistically significant differences (P<0.05). In the meantime, TNF-α levels in the grasper group were also lower than in the retractor group at both 24 hours and 48 hours postoperatively, with statistically significant differences observed only at 48 hours postoperatively (P<0.05) Table-III.

**Table-III:** Comparison of inflammatory factor levels between the two groups (*x̄*±*s*).

Indicator	Time Point	Grasper Group (n=198)	Retractor Group (n=333)	t value	P value
IL-6 (pg/mL)	1 day preoperatively	3.94 ± 1.89	4.02 ± 1.84	0.465	0.642
	24 hours postoperatively	7.90 ±3.11	12.02 ±4.70	10.997	<0.001
	48 hours postoperatively	5.91 ± 3.09	9.51± 4.66	9.676	<0.001
TNF-α (pg/mL)	1 hour preoperatively	10.78 ± 3.35	10.22 ± 3.64	0.819	0.413
	24 hours postoperatively	22.42± 3.78	22.52 ± 3.88	0.316	0.752
	48 hours postoperatively	14.44± 3.58	15.91 ± 3.83	4.369	<0.001
CRP (mg/L)	1 day preoperatively	3.75± 2.50	3.65± 2.51	0.416	0.678
	24 hours postoperatively	8.45± 3.64	11.41 ± 4.27	8.151	<0.001
	48 hours postoperatively	12.44± 3.66	15.43± 4.31	8.165	<0.001

In terms of early complications, the grasper group reported three cases of scrotal emphysema/hematoma (1.52%) and one case of incision infection (0.51%). By contrast, the retractor group had five cases of scrotal emphysema/hematoma (1.50%) and two cases of incision infection (0.60%). In this regard, the overall incidence of complications between the two groups (2.02% for the grasper group vs. 2.10% for the retractor group) exhibited no statistically significant differences (χ²=0.04, P=0.949). All children completed at least 12 months of follow-up (a median follow-up of 18 months). As for late complications, the grasper group had one case of recurrence (0.51%) and no cases of testicular atrophy or iatrogenic cryptorchidism, while the retractor group reported two cases of recurrence (0.60%), with no other late complications. Moreover, the comparison of recurrence rates between the two groups indicated no statistically significant differences (P>0.05).

## DISCUSSION

In this study, the comparison of needle graspers and mobile retractors as auxiliary instruments revealed differences in surgical efficiency and minimally invasive experience under the premise of maintaining equivalent safety. Therefore, this conclusion provides empirical evidence for the selection of individualized clinical strategies. The study indicated that the mean operation time in the grasper group was markedly shorter by 4.3 minutes than that in the retractor group (27.74 minutes vs. 29.94 minutes). This difference results from the fundamentally distinct operational principles of the two instruments. Specifically, the needle grasper demonstrates active and direct dissection (both blunt and sharp) and its slender design allows for precise grasping of the lateral peritoneum of the hernia sac neck and performs blunt dissection between the spermatic structures and the peritoneum, directly targeting the surgical site.^6^ This finding is consistent with a previous study indicating that the use of needle graspers is helpful for shortening operation time.^7^ By contrast, the mobile retractor can only perform sharp dissection, which may lead to significantly prolonged operation time or require additional incisions for the insertion of graspers to achieve high ligation of the hernia sac in cases of large hernia sacs or when scar tissue from previous incarcerations occurs.^8^

Some studies have also confirmed that satisfactory exposure could be achieved using mobile retractors in single-port laparoscopy.^9^ Therefore, the simplicity of the procedural steps may be considered an advantage of the graspers in efficiency. Furthermore, it is worth noting that the operation times for both groups in this study were significantly lower than those reported in several comparative studies of laparoscopy vs. open surgery (typically 25-40 min). This demonstrates that regardless of the auxiliary instruments used, laparoscopic techniques inherently boast a remarkable advantage in efficiency.^10^-^12^

Minimally invasive surgery is fundamentally characterized by its ability to mitigate bodily trauma and stress. The choice of instruments may affect the bodily pain response and inflammatory stress levels. This study revealed significantly lower pain scores at 24 hours postoperatively in the grasper group than in the retractor group (3.26 vs. 3.99). Additionally, the study also suggested that the grasper group had significantly lower pain scores and inflammatory factor levels (IL-6, CRP) at 24 hours postoperatively than the retractor group, which provides objective biological evidence that needle graspers cause less trauma. Specifically, serum IL-6 and CRP are classic indicators of tissue damage and surgical stress. According to the dynamic monitoring in this study, while inflammatory factor levels increased in both groups postoperatively, the grasper group showed significantly lower peak levels, suggesting a milder systemic inflammatory response.

This finding is corroborated by research in other fields, where a lighter inflammatory response is directly associated with reduced postoperative pain. Particularly, the force applied by the grasper is concentrated on the area of the hernia sac neck, resulting in less blunt traction and compression on the peritoneum and surrounding nerve plexus. By contrast, blunt dissection cannot be achieved using the mobile retractor and in challenging situations, it poses risks of penetrating the hernia sac and causing extraperitoneal bleeding, which may trigger a broader inflammatory response in the tissues, leading to an increased subjective perception of pain.^13^,^14^ This study confirmed the dual advantages of needle graspers in promoting rapid postoperative recovery through the combination of subjective pain scores with objective inflammatory factors.

Meanwhile, in terms of core safety and efficacy indicators in surgeries, this study also confirmed a high degree of equivalence between the two instruments. Specifically, no statistically significant differences were observed in intraoperative blood loss, early complications (e.g., scrotal hematoma and incision infection), or long-term recurrence rates between the two groups. This indicates that either instrument can safely and reliably achieve the ultimate goal of high ligation of the hernia sac neck. The overall recurrence rates in this study (0.51% for the grasper group vs. 0.60% for the retractor group) fall within the lower range reported by excellent centers at home and abroad (0%-2.4%)^15^ and are comparable to previous studies that report a 0% recurrence rate for laparoscopy.^16^ Furthermore, no differences were observed in the detection rate of contralateral occult hernias (about 14-16%) between the two groups, which collectively reflects the inherent advantages of laparoscopic techniques over open surgery.

Currently, the technical development of laparoscopy for pediatric hernia demonstrates two trends: 1. Further minimization of the surgical approach; 2. The specialization of instruments and standardization of procedures.^17^ Both needle graspers and mobile retractors have demonstrated significant application value in single-port laparoscopy.^18^,^19^ Despite needle graspers requiring a higher level of spatial perception and fine operation skills from the surgeons, their efficient, direct and low-damage style makes them an ideal tool for experienced surgeons who are in pursuit of surgical fluidity. Moreover, improvements in hernia repair instruments themselves also have an impact on surgical outcomes. In this regard, research has indicated that modified single-groove hernia needles can reduce the incidence of peritoneal injury and postoperative induration of puncture sites.^20^ This suggests that matching and optimizing auxiliary exposure instruments (graspers or retractors) with core ligation instruments (hernia needles) is an essential direction for enhancing overall surgical quality in the future.

### Limitations:

This study involves a single-center retrospective analysis and is inevitably subject to selection bias despite the relatively large sample size. All surgeries were performed by the same high-level team and caution shall be exercised when generalizing these findings to physicians with various levels of experience. Additionally, a stratified analysis of the learning curve of surgeons was not carried out, which is crucial for assessing the applicability of the instruments. Given this, prospective, multi-center randomized controlled trials shall be performed in the future, so as to further confirm these conclusions and refine the investigation of the performance differences between the two instruments in cases of varying difficulties (e.g., recurrent hernias, incarcerated hernias).

## CONCLUSIONS

Both needle graspers and mobile retractors-assisted LHHS are safe and effective minimally invasive techniques in the treatment of pediatric indirect inguinal hernias. Considering multiple dimensions of evidence, including operation time, pain control and inflammatory response, needle grasper-assisted LHHS demonstrates superior minimally invasive performance and recovery quality under the premise of achieving the same level of surgical safety.

### Author’s Contributions:

**HJ:** Study design, literature search and manuscript writing, is responsible and accountable for the accuracy or integrity of the work.

**RW** and **ZD:** Were involved in data collection, data analysis and interpretation. Critical Review.

**BW** and **SZ :** Was involved in the manuscript revision and validation.

All authors have read and approved the final manuscript.
